# Therapeutic Potential of Induced Neural Stem Cells for Parkinson’s Disease

**DOI:** 10.3390/ijms18010224

**Published:** 2017-01-22

**Authors:** Dong-Hee Choi, Ji-Hye Kim, Sung Min Kim, Kyuree Kang, Dong Wook Han, Jongmin Lee

**Affiliations:** 1 Department of Medical Science, School of Medicine, Konkuk University, 1 Hwayang-dong, Gwangjin-gu, Seoul 143-701, Korea; dchoi@kku.ac.kr; 2 Center for Neuroscience Research, Institute of Biomedical Science and Technology, Konkuk University, 1 Hwayang-dong, Gwangjin-gu, Seoul 143-701, Korea; hljf2326@hanmail.net; 3 Department of Stem Cell Biology, School of Medicine, Konkuk University, 1 Hwayang-dong, Gwangjin-gu, Seoul 143-701, Korea; mins0424@konkuk.ac.kr (S.M.K.); krkang@konkuk.ac.kr (K.K.); 4 Konkuk Univesity Open-Innovation Center, Institute of Biomedical Science & Technology, Konkuk University, 1 Hwayang-dong, Gwangjin-gu, Seoul 143-701, Korea; 5 Department of Advanced Translational Medicine, School of Medicine, Konkuk University, 1 Hwayang-dong, Gwangjin-gu, Seoul 143-701, Korea; 6 Department of Rehabilitation Medicine, Konkuk University School of Medicine, 1 Hwayang-dong, Gwangjin-gu, Seoul 143-701, Korea

**Keywords:** Parkinson’s disease, induced neural stem cell, differentiation, reprogramming, transplantation, functional recovery

## Abstract

Parkinson’s disease (PD) is a chronic, neurodegenerative disorder that results from the loss of cells in the substantia nigra (SN) which is located in the midbrain. However, no cure is available for PD. Recently, fibroblasts have been directly converted into induced neural stem cells (iNSCs) via the forced expression of specific transcription factors. Therapeutic potential of iNSC in PD has not been investigated yet. Here, we show that iNSCs directly converted from mouse fibroblasts enhanced functional recovery in an animal model of PD. The rotational behavior test was performed to assess recovery. Our results indicate that iNSC transplantation into the striatum of 6-hydroxydopamine (6-OHDA)-injected mice can significantly reduce apomorphine-induced rotational asymmetry. The engrafted iNSCs were able to survive in the striatum and migrated around the medial forebrain bundle and the SN pars compacta. Moreover, iNSCs differentiated into all neuronal lineages. In particular, the transplanted iNSCs that committed to the glial lineage were significantly increased in the striatum of 6-OHDA-injected mice. Engrafted iNSCs differentiated to dopaminergic (DA) neurons and migrated into the SN in the 6-OHDA lesion mice. Therefore, iNSC transplantation serves as a valuable tool to enhance the functional recovery in PD.

## 1. Introduction

Parkinson’s disease (PD) is one of the most common neurodegenerative diseases and is associated with motor and non-motor dysfunction. PD involves loss of dopaminergic (DA) neurons in the substantia nigra (SN) [1]. The currently established therapeutic approaches, which involve drug treatment [2,3] and deep-brain stimulation [4], can temporarily ameliorate symptoms, but cannot cure the disease. Thus, over the last two decades, cell replacement therapy has proven, at least experimentally, to be a potential treatment in patients with PD [5,6,7,8,9,10] and animal models of PD [11,12,13,14,15,16,17,18]. Therefore, cell-based therapy has been proposed as a promising treatment strategy for PD. In this context, some researchers have used adult bone marrow-derived mesenchymal stem cells [19,20] and olfactory ensheathing cells [21], but these cells have restricted capacity to differentiate to DA neurons.

Despite the therapeutic potential of in vivo derived neural stem cells (NSCs), their restricted accessibility as well as potential immune problem from their allogenic application might block their clinical application [1]. Thus, the patient-derived induced pluripotent stem cells (iPSCs) have been long considered as a promising cell source for autologous cell therapy due to their unlimited differentiation potentials as well as self-renewal capacity. However, there are still several huddles precluding their translation into clinical practice such as the potential risk of tumor formation by the residual undifferentiated iPSCs upon transplantation [22,23,24]. Since the recent direct conversion technology using lineage specific transcription factors do not necessarily undergo an iPSC state, the potential risk of tumor formation can be minimized or eliminated by using this brand new technology.

Previously, we and other groups generated self-renewable and functional induced NSCs (iNSCs) from easily accessible somatic fibroblasts using the direct conversion technology [25,26]. Directly converted iNSCs closely resemble the in vivo counterparts in terms of the gene expression pattern, epigenetic status, and both in vitro and in vivo differentiation potential [27]. Although iNSCs have been considered to be a feasible, effective, and autologous source for clinical applications, their therapeutic ability has not been addressed fully. In the current study, we investigated the therapeutic potential of iNSCs in a mouse model of PD. Engrafted iNSCs differentiated into all neuronal lineages, including specific DA neurons, which resulted in the recovery of motor functions. Overall, these data suggest that striatal transplanted iNSCs may have therapeutic potential for PD.

## 2. Results

### 2.1. Direct Conversion of Fibroblasts into iNSCs

**Figure 1:**
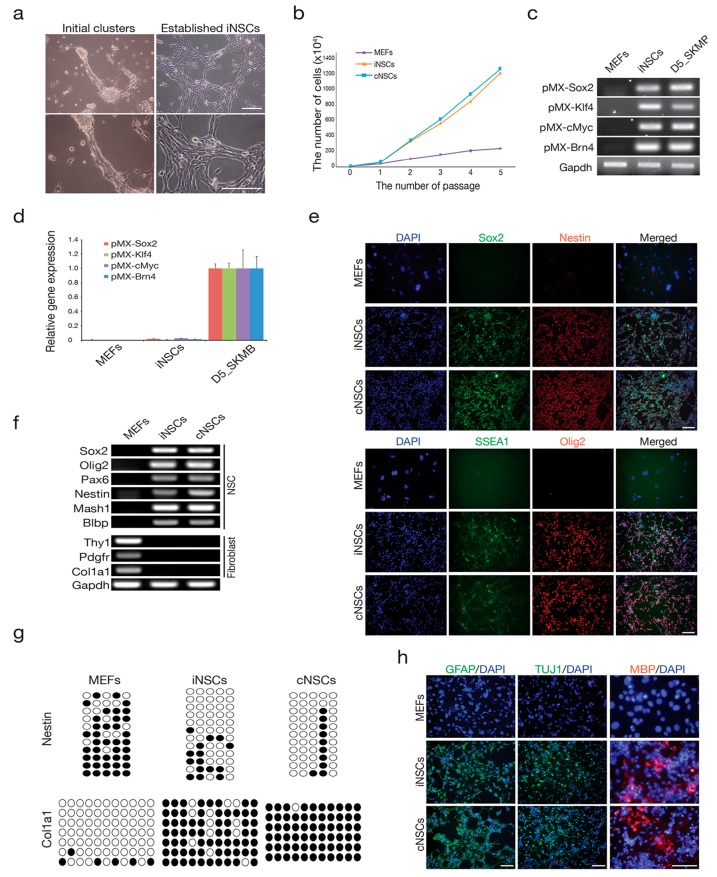
Direct conversion of fibroblasts into induced neural stem cells (iNSCs) (**a**) Morphology of the initial iNSC clusters at four weeks after induction of reprogramming (left) and established iNSC cell lines at passage 10 (right). Scale bars = 100 µm; (**b**) proliferation of iNSCs. Cells (1 × 10^5^) were passaged every two days on the wells of 12-well plates; (**c**) iNSCs showed integration of retroviral transgenes encoding *Sox2*, *Klf4*, *cMyc*, and *Brn4* (SKMB); (**d**) expression levels of retroviral transgenes in the iNSCs at passage 15. The expression levels are normalized to those of mouse embryonic fibroblasts (MEFs). Error bars indicate the standard derivation of triplicate values. d5_SKMB, Day 5 after SKMB retroviral transduction; (**e**) immunofluorescence microscopy images of iNSCs and control NSCs (cNSCs) using antibodies against Sox2/Nestin (upper panel) and Olig2/SSEA1 (lower panel). MEFs and cNSCs were used as the negative and positive control, respectively. Scale bars = 100 µm. (**f**) RT-PCR analysis of markers for NSCs and fibroblasts in the established iNSC line; (**g**) the DNA methylation status on the second intron of Nestin and the promoter region of Col1a1 in MEFs, cNSCs, and iNSCs was assessed by bisulfite sequencing PCR. Open and filled circles represent unmethylated and methylated CpGs, respectively; (**h**) in vitro differentiation potential of iNSCs into astrocytes, neurons, and oligodendrocytes, as shown by immunostaining with antibodies against glial fibrillary acidic protein (GFAP), Class III β-tubulin (Tuj-1), and myelin basic protein (MBP), respectively. Scale bars = 100 µm.

To generate iNSCs, the genes that encode four transcription factors (*Brn4*, *Sox2*, *Klf4*, and *c-Myc*) were introduced into mouse embryonic fibroblasts (MEFs) that were derived from mouse embryos after carefully removing the head, spinal cord, and all internal organs, as described in our previous studies [25,26,28,29,30]. Similar to that reported in those studies, the initial iNSC clusters that were normally observed after three to four weeks of transduction quickly became dominant after serial passaging (two to three times) and eventually became a stably self-renewing iNSC line (Figure 1a,b). All four transgenes were integrated in the iNSC genome, but their expression was silenced in the established iNSCs (Figure 1c,d). The established iNSCs expressed all of the NSC markers examined at both the messenger RNA (mRNA) and protein levels (Figure 1e,f). In contrast, the expression of fibroblast markers was completely repressed in the established iNSCs (Figure 1f). In the line for which expression profiling was performed, the regulatory region of *Col1a1*, which is a fibroblast marker, became highly methylated in iNSCs to a level that was similar to that of control NSC (cNSCs) (Figure 1g). Conversely, the second intron of *Nestin*, which is an NSC marker, was completely demethylated in iNSCs (Figure 1g), indicating that iNSCs were reprogrammed into an NSC-like state even at the epigenetic level.

To address the presence of functional stemness in the established iNSCs, we examined the tripotential differentiation capacity of iNSCs by inducing in vitro differentiation. Under specific differentiation conditions, iNSCs successfully differentiated into neurons, astrocytes, and oligodendrocytes, as determined by immunostaining using antibodies against neuron-specific Class III β-tubulin (Tuj-1), glial fibrillary acidic protein (GFAP), and myelin basic protein (MBP), respectively (Figure 1h). Taken together, our data indicate that the directly converted iNSCs acquired neural stemness not only at the molecular level, but also at the functional cellular level, to levels similar to that observed for control NSCs from brain tissues.

### 2.2. Engrafted iNSCs Could Differentiate into All Neuronal Lineages

**Figure 2:**
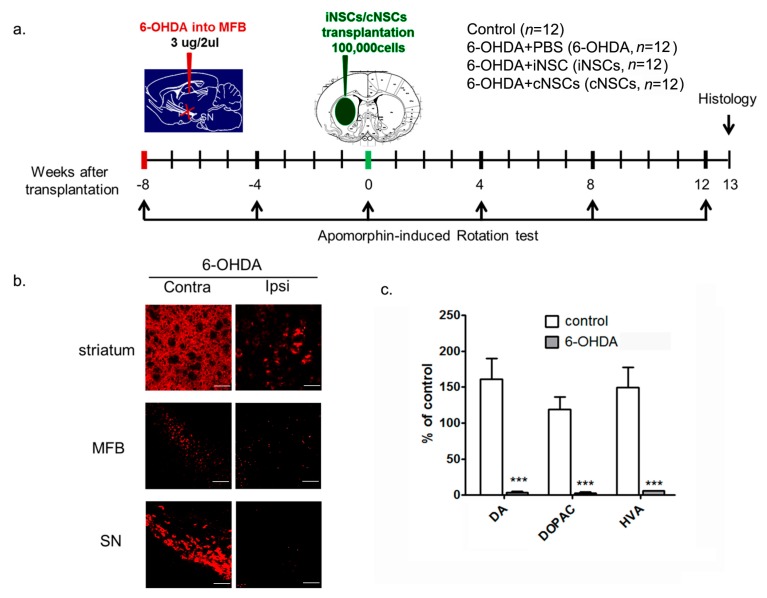
The timeline of the experiment and animal models. (**a**) Three micrograms of 6-hydroxydopamine (6-OHDA) were unilaterally injected into the medial forebrain bundle (MFB). Eight weeks later, saline (6-OHDA), induced neural stem cells (iNSCs, 6-OHDA + iNSCs), or control neural stem cells (cNSCs, 6-OHDA + cNSCs) were injected into the ipsilateral striatum. Control, 6-OHDA, 6-OHDA + iNSCs, and 6-OHDA + cNSCs mice were evaluated using the apomorphine-induced rotation behavior test at intervals of four weeks after 6-OHDA injection. All animals were used for behavioral analysis and histological studies; (**b**) representative photomicrographs of tyrosine hydroxylase (TH) staining in the mouse striatum, MFB, and SN sections. Three micrograms of 6-OHDA were injected into the MFB. Eight weeks later, DA neurons in the striatum, MFB, and substantia nigra (SN) were visualized with TH immunostaining. Scale bars = 50 µm; (**c**) dopaminergic (DA), 3,4-dihydroxyphenylacetic acid (DOPAC), and homovanillic acid (HVA) levels were determined in the stratum at eight weeks after 6-OHDA injected into the MFB. Results are presented as the mean ± SEM, *n* = 6. *** *p* < 0.001 vs. control.

To assess the differentiation potential of iNSCs, we transplanted 1 × 10^5^ iNSCs into the ipsilateral striatum of mice carrying a 6-hydroxydopamine (6-OHDA) unilateral lesion eight weeks after injury. A detailed timeline for the experiment is provided (Figure 2a). Tyrosine hydroxylase (TH)-positive DA neurons almost disappeared in the striatum, medial forebrain bundle (MFB), and SN pars compacta (SNpC), and the levels of DA and DA metabolites 3,4-dihydroxyphenylacetic acid (DOPAC), and homovanillic acid (HVA) were significantly decreased in the striatum at eight weeks after 6-OHDA injection (Figure 2b,c).

**Figure 3:**
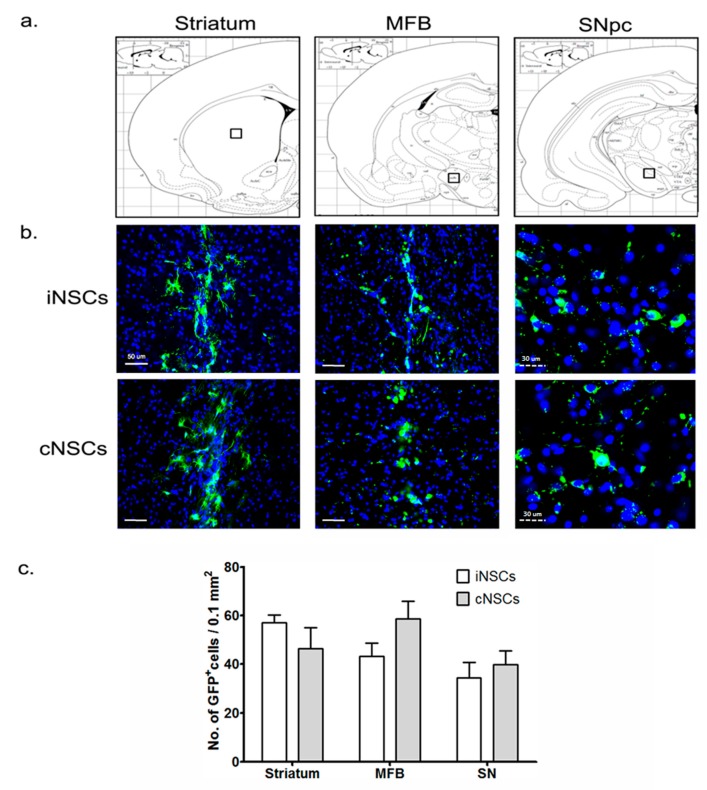
Identification and migration in the striatum, MFB, and SN pars compacta (SNpC) of grafted murine cNSCs and iNSCs (**a**,**b**) Representative photomicrograph of green fluorescent protein (GFP)-positive engrafted cNSCs or iNSCs in the transplantation site (striatum), injury sites (MFB), and SNpC indicated the square boxes at 12 weeks post-transplantation. Scale bars: solid line = 50 μm and dotted line = 30 μm; (**c**) GFP-positive cells were counted in the striatum, MFB, and SNpC. Blue colors show Topro-3 nuclear marker. Results are presented as GFP^+^ cell counts per 0.1 mm^2^ areas (*n* = 6).

**Figure 4:**
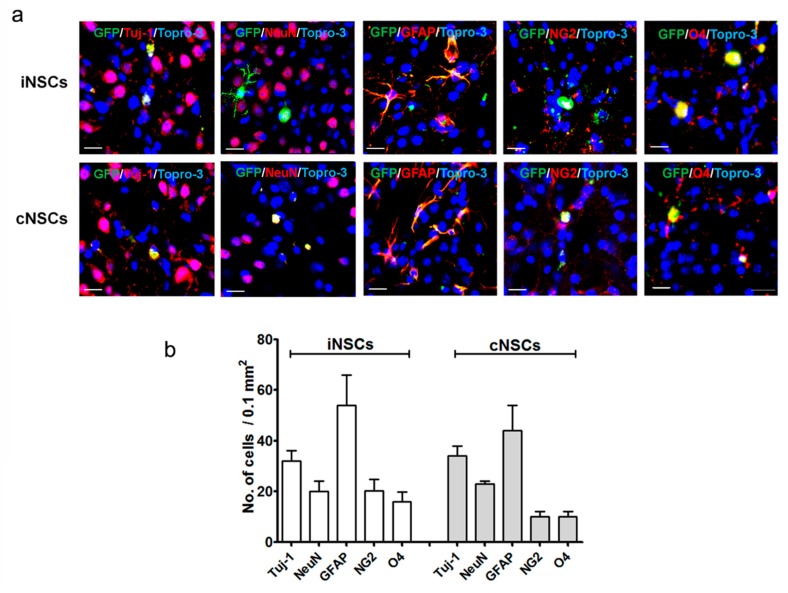
Differentiation into all neuronal lineages of engrafted iNSCs (**a**) Representative photomicrograph of GFP-positive iNSCs and cNSCs differentiated into both the neuronal and the glial lineages in vivo as determined by co-staining with Tuj-1 or NeuN (red) for neurons, GFAP or NG2 for astrocytes, and O4 for oligodendrocytes, respectively in the striatum at 12 weeks after transplantation. Blue colors show Topro-3 nuclear marker. Scale bars = 20 µm; (**b**) results are presented as triple stained cell counts per 0.1 mm^2^ areas (*n* = 6).

Phosphate-buffered saline (PBS) and cNSCs were used as negative and positive controls, respectively. We detected green fluorescent protein (GFP)-positive cells, to identify the grafted cNSCs and iNSCs (Figure 3). At 12 weeks after transplantation, grafted murine cNSCs and iNSCs had migrated into the MFB and SNpC, as well as into the striatum (Figure 3a,b). The numbers of GFP-positive iNSCs in the striatum, MFB, and SNpC was not differ from those obtained for cNSCs (Figure 3c). Transplanted iNSCs and cNSCs were positive for the neuronal markers Tuj-1 and a neuronal specific nuclear protein (NeuN), indicating that the grafted cells had committed to the neuronal lineage in vivo (Figure 4a,b).

Furthermore, the grafted cells had also committed to the glial lineage, as evidenced by the presence of GFP^+^/GFAP^+^ and GFP^+^/NG2^+^ (neural/glial antigen 2) cells (Figure 4c,d). Finally, the transplanted iNSCs also exhibited the ability to differentiate into oligodendrocytes, as evidenced by the presence of GFP^+^/oligodendrocyte marker O4 (O4)^+^ cells (Figure 4e,f). Taking all these results together, we conclude that iNSCs and cNSCs have the potential to undergo differentiation in all neural cell lineages. Our data show that the differentiation efficiency of iNSCs into neurons, glial cells, and oligodendrocytes is comparable with that of cNSCs (Figure 4).

### 2.3. Engrafted iNSCs Differentiated to DA Neurons and Migrated into the SN

**Figure 5:**
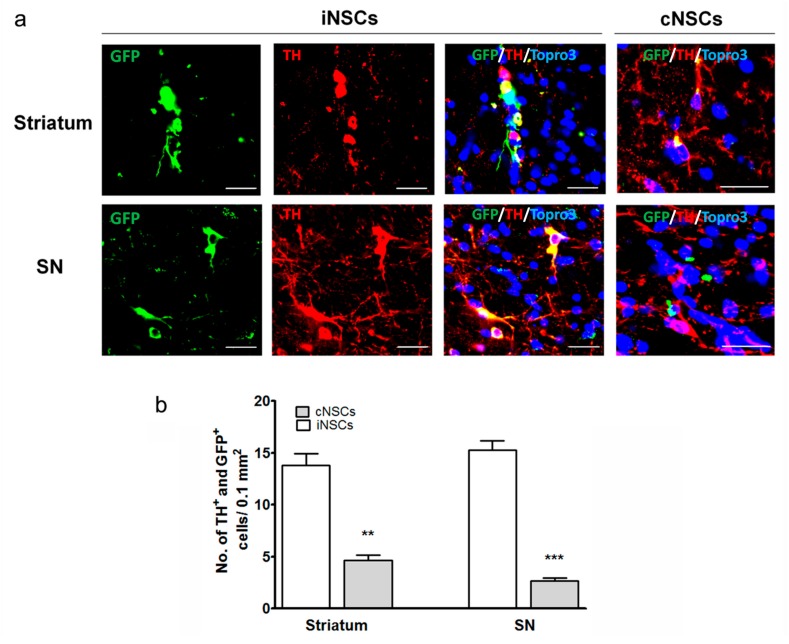
DA differentiation of engrafted iNSCs (**a**) Representative photomicrograph of GFP-positive iNSCs and cNSCs (green) differentiated into DA neurons in striatum and SNpC as determined by co-staining with TH (red). Blue colors show Topro-3 nuclear marker. Scale bars = 30 µm; (**b**) results are presented as triple stained cell counts per 0.1 mm^2^ areas. ** *p* < 0.01, *** *p* < 0.001 vs. cNSCs (*n* = 6).

To understand whether one of the subtypes of the transplanted NSCs that survived and differentiated to neurons became DA neurons in vivo, the brain tissues were stained with the tyrosine hydroxylase (TH) antibody, which is specific to DA neurons. Some of the neurons were costained with GFP and TH, indicating that the engrafted cells differentiated into DA neurons (Figure 5) in the striatum. Furthermore, migration of GFP^+^/TH^+^ cells into the SN was observed. Compared with the cNSC group, the iNSCs group exhibited an increased number of GFP^+^/TH^+^ cells in the striatum and SN at 12 weeks after stem cell transplantation (Figure 5). Statistical analysis revealed a significant increase of these cells in the iNSCs group compared with the cNSC group (Figure 5, ** *p* < 0.01). These findings may be one of the potential reasons why iNSCs resulted in enhanced behavioral improvement as compared to cNSCs.

### 2.4. Engrafted iNSCs Improved Motor Performance in the 6-OHDA-Induced PD Mouse Model

**Figure 6:**
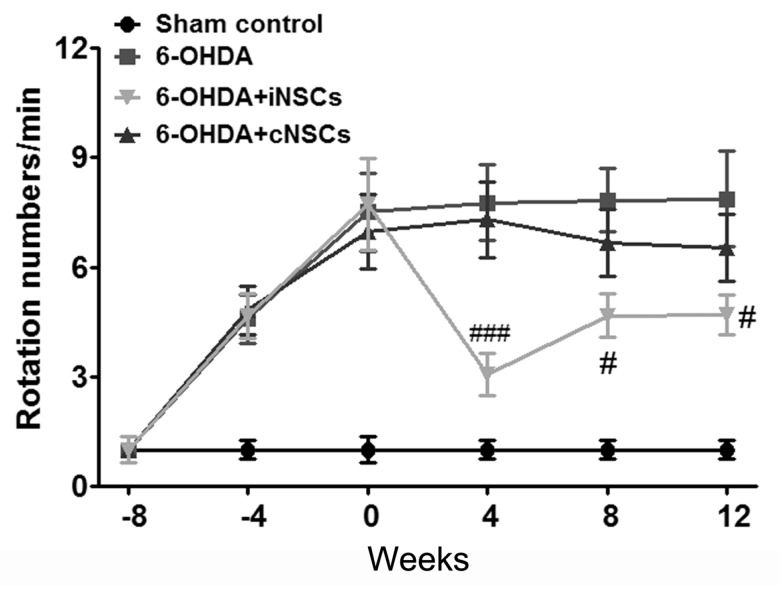
Engrafted iNSCs improved motor performance in the 6-OHDA-induced PD mouse model (a) Total apomorphine-induced rotation numbers (0.5 mg/kg) were counted at 4, 8, and 12 weeks after cell injection. iNSCs (*n* = 12) injected group showed the improved symptom compared to PBS injected groups (*n* = 12), while average rotation score of the iNSC group was decreased compared to the cNSC injected group. Each value depicts mean ± SEM of number of rotation. # *p* < 0.05, ### *p* < 0.001 vs. 6-OHDA.

In apomorphine-induced rotation test [31], PBS, iNSCs, and cNSCs were injected at eight weeks after 6-OHDA injection, a time point at which contralateral rotations were over 7 turns/min. The rotational scores are shown in Figure 6.

**Figure 7:**
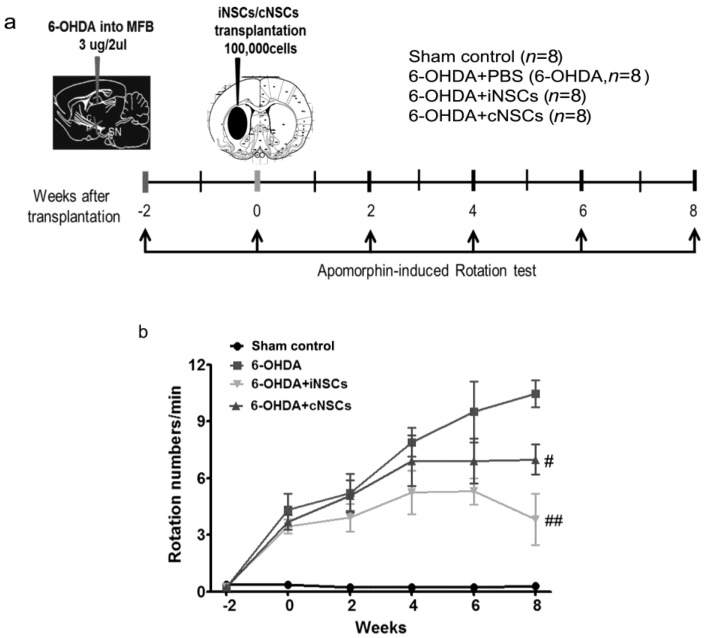
Engrafted iNSCs protect the motor dysfunction increased in the 6-OHDA-induced PD mouse model (**a**) Total apomorphine-induced rotation numbers (0.5 mg/kg) were counted at two, four, six, and eight weeks after iNSCs and cNSC injection; (**b**) each value is presented as mean ± SEM of number of rotation. # *p* < 0.05, ## *p* < 0.01 vs. 6-OHDA (*n* = 8).

The 6-OHDA control mice (6-OHDA), which received injections of PBS into their dopamine-denervated striata, did not show any improvement. In contrast, the iNSC-injected PD mice showed a significantly decreased number of rotations/min (F_(3,44)_ = 6.91; *p* < 0.05 vs. 6-OHDA) at 4, 8, and 12 weeks posttransplantation. These results indicate that iNSC transplantation improved motor performance in the 6-OHDA-induced PD mouse. There was no statistical difference between 6-OHDA + cNSC and 6-OHDA mice. To determine the effects of iNSCs and cNSCs in moderate-damage conditions, PBS, iNSCs, and cNSCs were injected at two weeks after 6-OHDA injection, at a time point when the contralateral rotations were less than 4 turns/min. The rotational response to apomorphine was examined at two, four, six, and eight weeks posttransplantation (Figure 7). The 6-OHDA control (6-OHDA) animals (*n* = 8) exhibited a gradual increase in rotational score, indicating progressive DA neuronal loss, whereas animals that carried iNSCs and cNSCs did not show a significant increase in apomorphine-induced rotations. Importantly, the decrease in rotational score was gradual (Figure 7), and animals that carried iNSCs (*p* < 0.01) or cNSCs (*p* < 0.05) showed a significant decrease in rotations vs. 6-OHDA control (6-OHDA) animals at eight weeks posttransplantation. Similar significant differences were obtained regarding measures of the percentage of change in rotations.

## 3. Discussion

In the present study, we found that iNSCs and cNSCs grafts improved the motor performance of PD mice via the transplantation of iNSCs and cNSCs that survived, migrated, and differentiated into neuronal and glial cells in the striatum and SNpC. In particular, transplanted iNSCs resulted in the predominant DA neuronal differentiation in the striatum and SNpC. Therefore, iNSCs and cNSCs may be a promising cell source for the treatment of PD.

The major pathology of PD is the progressive degeneration of DA neurons located in the SN in the midbrain, which send axonal projections to the striatum and are involved in the circuits that control motor functions [32].

Fetal ventral mesencephalon (fVM) tissue transplants were first performed to evaluate the efficacy and safety of fVM in the initial open-label trials on PD treatment [32]. However, the next two double-blind trials sponsored by National Institutes of Health failed in improving patient outcomes [32]. A retrospective analysis revealed several possible reasons for these results, including patient selection, the heterogeneity of grafts, immune recognition of the grafts, lack of standardization of the transplantation procedure, and uneven distribution of the grafts [32]. Moreover, graft-induced dyskinesia is another side effect that should be considered [32,33,34,35,36,37]. Advances in reprogramming technologies have been reported in recent years, which may provide solutions to the problems associated with fVM tissues. For this purpose, several types of primary adult stem cells (or progenitors), such as iPSC-derived DA cells, induced DA neurons (iDAs), and iNSCs, have been transplanted into the PD model [32].

Using three or four factors, we generated iNSCs from mouse fibroblasts [25,29,30]. These iNSCs can self-renew and differentiate into different neuronal subtypes, including DA neurons. To define the therapeutic potential of these iNSCs, we showed previously that iNSCs directly converted from mouse fibroblasts on spinal cord injury (SCI) animals [28]. Engrafted iNSCs in spinal cords of SCI animals differentiated into all neuronal lineages, including diverse subtypes of mature neurons [28]. Moreover, iNSC-derived neurons shaped neurotransmitters with host neurons, hence improving the locomotor function restoration [28]. Compared with iPSCs, iNSCs present a lower tumorigenic risk. However, retroviral vector-mediated gene delivery for generating iNSCs may cause insertional mutagenesis into the host genome. Therefore, in order to bypass the undesired side effects of using retroviral vector-mediated gene delivery, we very recently described the integration-free direct conversion protocol using episomal (extrachromosomal) vector system [29]. However, the current study is a proof-of-concept study for verifying the therapeutic potential of iNSCs for PD treatment and thus, we did not employ the integration-free iNSC line in this study. In our previous study [29], we were able to confirm that the integration-free iNSCs from episomal vectors are indistinguishable from iNSCs generated by retroviral vector system in terms of their self-renewal capacity, gene expression patterns, epigenetic status, electrophysiological activity, and both in vitro and in vivo differentiation potentials. Therefore, the therapeutic potentials of iNSCs from distinct generation protocols (retroviral system vs. episomal vector system) might be comparable.

Furthermore, we previously also performed the long-term monitoring of transplanted iNSCs. We have previously demonstrated that none of the retrovirus-mediated iNSC transplanted animals (out of 13 mice and 28 rats) showed any tumor formation up to a maximum of six months after transplantation [26,28,38]. Thus, the safety of retrovirus-mediated iNSCs has been proved. In the current study, we also traced the fate of engrafted iNSCs up to 12 weeks of transplantation and found that the engrafted iNSCs could differentiate into both the neuronal and glial lineages without forming tumors. Another group also demonstrated that iNSCs that were converted by the introduction of eight transcription factors [39] and overexpressed Lmx1a, which is a key determinant in the specification of DA neurons (iNSC-Lmx1a), enhanced DA neuron production from iNSCs in vitro [40]. Those iNSC-Lmx1a cells improved the behavioral performance of PD mice. Although the previous study successfully demonstrated the generation of iNSCs which could be readily differentiated into DA neurons using a total of nine factors including Lmx1a, it is hard to conclude which cell line is the better cell source for treating PD because we have not compared both cell types side-by-side.

Besides survivability and neuronal differentiation of grafted iNSC, we observed a significant improvement in the apomorphine-induced rotation in iNSC-transplanted mice. This significant improvement appeared from four weeks posttransplantation (Figure 6). Moreover, diminishing DA neuronal loss appeared after posttransplantation, suggesting neuroprotection against progressive DA degeneration induced by 6-OHDA in the nigrostriatal tract (Figure 7). Engrafted iNSCs differentiated to neuronal and glia cells (Figure 4). The neurons that resulted from the differentiation of iNSCs included dominantly DA neurons that migrated from the striatum into the SNpC (Figure 5). These findings may be one of the potential reasons why iNSCs resulted in enhanced behavioral improvement as compared to cNSCs.

Therefore, treatment of PD with iNSCs is a promising direction in the foreseeable future.

## 4. Materials and Methods

### 4.1. Cell Culture

Mouse embryonic fibroblasts (MEFs) were derived at embryonic day 13.5 after carefully removing the head and all the internal organs including spinal cord. MEFs were maintained in dulbecco’s modified eagle’s medium (DMEM) (Biowest, Riverside, MO, USA) containing 10% fetal bovine serum (FBS, Biowest), 5 mL of penicillin/streptomycin/glutamine (Invitrogen, Waltham, MA, USA), and 5 mL of minimum essential medium non-essential amino acids (MEM NEAA) solution (Invitrogen) in 500 mL of MEF medium. The control NSCs were isolated from brain of fetus at embryonic day 13.5. The control NSCs and established iNSCs were maintained in NSC culture medium: DMEM/F-12 supplemented with 10 mL of B27 supplements (Gibco, Waltham, MA, USA), 10 ng/mL epidermal growth factor (EGF, Peprotech, Rocky Hill, NJ, USA), 10 ng/mL (basic fibroblast grwoth factor (bFGF, Peprotech), and 5 mL of penicillin/streptomycin/glutamine (Invitrogen) in 500 mL of NSC medium.

### 4.2. Generation of iNSCs

To generate iNSCs, the MEFs were transduced with retroviral particles and cultured as previously described [25,29,30]. Briefly, 5 × 10^4^ fibroblasts were plated onto the gelatin-coated 35-mm dish and incubated with ecotropic retroviruses for 48 h. After 48 h of incubation, the medium containing retroviral particles was replaced with NSC medium. In order to enrich the initial cluster of iNSCs, non-reprogrammed fibroblasts or unwarranted cells were removed with a cell scraper as previously described [25]. The initial iNSC clusters were observed around four weeks after induction of reprogramming process. The clusters were maintained for two to three more days for maturation, and then passaged into 1:1 ratio for the establishment of iNSCs.

### 4.3. Gene Expression Analysis by RT-PCR and qPCR

Total RNA was isolated using the Hybrid-RTM kit (GeneAll, Seoul, Korea), and 1 μg of total RNA was reverse transcribed into cDNA using the high capacity cDNA reverse transcription kit (Applied Biosystems, Waltham, MA, USA) according to the manufacturer’s instructions. RT-PCR was performed using the GoTaq green master mix (Promega, Madison, WI, USA). qPCR was performed using SYBR green PCR Master Mix (Applied Biosystems, Waltham, MA, USA) on the ABI 7500 real-time PCR system (Applied Biosystems). Δ*C*t values were calculated by subtracting *GAPDH C*_t_ value from that of target genes. Relative expression levels were calculated by using 2^−ΔΔ*C*t^ method. PCR primer sequences are presented in Table 1.

### 4.4. DNA Methylation Analysis

To analyze the DNA methylation status in both iNSCs and cNSCs, sodium bisulfite was treated using EpiTect Bisulfite Kit (QIAGEN, Valencia, CA, USA) according to the manufacturer’s instructions. Briefly, unmethylated cytosine nucleotides in genomic DNA were converted into uracil nucleotides upon sodium bisulfite treatment. To obtain enough PCR product, two rounds of PCR were performed using SuperTaq (Ambion, Waltham, MA, USA) according to our previous reports [28,41]. Three microliters of product from the first PCR reaction were used as template for the second round of PCR. The PCR amplicants were subcloned into the pCR2.1-TOPO TA vector (Invitrogen). The reconstructed plasmid DNA was isolated using the QIAprep Spin Miniprep Kit (QIAGEN) and purified plasmids were individually sequenced (Macrogen, Seoul, Korea). Clones were analyzed using QUMA software (http://quma.cdb.riken.jp).

### 4.5. Immunocytochemistry

The cells were fixed with 4% paraformaldehyde (Sigma-Aldrich, St. Louis, MO, USA) for 20 min at room temperature, and then blocked with Dulbecco’s phosphate-buffered saline (DPBS) (Biowest) containing 0.3% Triton X-100 (Sigma-Aldrich) and 5% FBS (Biowest) for 2 h at room temperature. The cells were then incubated with primary antibodies at 4 °C for 16 h, washed three times with DPBS, and then incubated with appropriate fluorescence-conjugated secondary antibody for 2 h at room temperature in the dark. Nuclei were stained with Hoechst33342 (Sigma-Aldrich). Primary antibodies used for immunofluorescence are as follows: mouse anti-Nestin (Millipore, Billerica, MA, USA, 1:200), goat anti-Sox2 (Santa Cruz Biotechnology, Santa Cruz, CA, USA, 1:200), mouse anti-SSEA1 (Santa Cruz Biotechnology, 1:100), rabbit anti-Olig2 (Millipore, 1:200), mouse anti-Tuj-1 (Covance, Princeton, NJ, USA, 1:500), rabbit anti-Glial fibrillary acidic protein (GFAP) (DAKO, Glostrup, Denmark, 1:500), and rat anti-Myelin basic protein (MBP, Abcam, Cambridge, MA, USA, 1:100).

### 4.6. In Vitro Differentiation

For differentiation into neurons, iNSCs were plated onto laminin/poly-lysine-coated dishes at 2.5 × 10^4^ cells per cm^2^ in NSC medium. The next day, medium was replaced with neural differentiation medium: DMEM/F-12 supplemented with 2% B27 supplements (Gibco), 1% penicillin/streptomycin/glutamine (Invitrogen), and 10 ng/mL bFGF (Peprotech) in 100 mL of neural differentiation medium. On day 4 of differentiation, the medium was changed into neural differentiation medium containing 200 mM ascorbic acid (Sigma-Aldrich) without growth factors for 8–10 more days. For differentiation into astrocytes, iNSCs were cultured in DMEM/F-12 supplemented with 10% FBS and 1% penicillin/streptomycin/glutamine on gelatin-coated dishes for five days. Finally, for differentiation into oligodendrocytes, iNSCs were plated onto laminin/poly-lysine-coated dishes at 2.5 × 10^4^ cells per cm^2^ in NSC medium. The next day, the medium was replaced with oligodendrocyte differentiation medium: DMEM/F-12 supplemented with 2% B27 supplements, 1% penicillin/streptomycin/glutamine, 10 ng/mL bFGF, and 10 ng/mL platelet-derived growth factor (PDGF, Sigma-Aldrich) in 100 mL of oligodendrocyte differentiation medium. On day 4 of differentiation, the medium was changed into oligodendrocyte differentiation medium containing 30 ng/mL T3 (Sigma-Aldrich) and 200 mM ascorbic acid for another four days. The differentiation medium was replaced with fresh medium every other day.

### 4.7. Animals

Since a recent meta-analysis of the world-wide data indicated that the prevalence of PD is more frequent in males than in females in the age group of 50–79 years [42,43], a total of 80 male C57/BL6 mice ((12 weeks, weighing 27–30 g; Samtako BioKorea. Co., Ltd., Osan, Korea; 20 sham control mice (Sham control) and 60 6-OHDA injected mice, 6-OHDA, Sigma-Aldrich)) were enrolled in this study and maintained in a room at 22 °C under a 12 h light/dark cycle; chow and water were provided ad libitum. The room was illuminated by incandescent lamps (luminous flux, 11.77 lumen). Animal treatments, including anesthesia and euthanasia, were carried out in accordance with the Principle of Laboratory Animal Care (NIH publication No. 85-23, revised 1985). All experimental procedures were approved by the Animal Experiment Review Board of Institutional Animal Care and Use Committee (IACUC) of Konkuk University (Permit Number: KU12035; permission date: 21 March 2012).

### 4.8. Animal Treatment and Unilateral 6-OHDA Lesion in MFB

Under ketamine (50 mg/kg) and xylazine (5 mg/kg) mixture through intraperitoneal (i.p.) injection, mice in the 6-OHDA group were fixed in a stereotaxic apparatus (Stoelting Co., Wood Dale, IL, USA), and then an incision was made on the scalp. The 6-OHDA solution (3 μg/2 μL 6-OHDA dissolved in 0.9% saline containing 0.01% ascorbic acid as antioxidants) was injected unilaterally into the MFB using a 10 μL Hamilton microsyringe at one site of MFB using the following coordinates according to the Allen Mouse Brain Atlas [44,45]: 1.2 mm anterior to the bregma; 1.1 mm lateral to the midline; and 5.0 mm below the dura mater. Injections were carried out at a rate of 0.2 μL /min and a volume of 2 μL was injected in the site. The needle was left at the injected sites for 5 min before being withdrawn. Mice in the control group received the same volume of saline in the same injected sites (*n* = 12/group).

### 4.9. Measurement of DA and DA Metabolites (DOPAC and HVA)

The levels of DA and its metabolites 3,4-dihydroxyphenylacetic acid (DOPAC), and homovanillic acid (HVA) were estimated from the isolated striatal homogenate using a high-performance liquid chromatography (HPLC) with electrochemical detection (ECD) system [46]. In brief, the dissected striatal tissues were homogenized with 0.1 M perchloric acid and centrifuged at 15,000× *g* for 15 min at 4 °C. Ten microliters of the supernatant were injected into a HPLC pump (Agilent Technologies 1200 Series, Santa Clara, CA, USA) having a reversed-phase C18 column (4.6 mm × 150 mm × 5 μm, Eclipse XDB-C18) connected to an ECD (Antec, Decade II, Zoeterwoude, The Netherlands). The mobile phase (pH 2.9) consisted of 75 mM NaH_2_PO_4_, 1.7 mM 1-octanesulfonic acid, 100 μL/L triethylamine, 25 μM EDTA, 9% acetonitrile, and 2 mM NaCl. The amounts of DA and its metabolites were calculated using Agilent ChemStation software (Agilent technologies, Santa Clara, CA, USA) and standard curve.

### 4.10. Transplantation

Before transplantation, the 5 × 10^5^ of cNSCs or iNSCs were transduced with ecotropic virus encoding GFP. The supernatant containing GFP virus was replaced with fresh NSC medium 24 h after transduction. To avoid serum-induced differentiation of cNSCs or iNSCs, GFP virus was concentrated by using Retro-X™ Concentrator (Takara Bio Inc., Kusatsu, Shiga, Japan) according to the manufacturer’s instructions. cNSCs or iNSCs (passage 20–30) containing GFP were stereotaxically injected into the striatum two weeks (*n* = 24) or eight weeks (*n* = 36) after 6-OHDA operation. All 6-OHDA operated mice were respectively allocated into three groups (two weeks, *n* = 8/group; eight weeks, *n* = 12/group), PBS, NSCs, iNSCs groups. Mice were deeply anesthetized via a ketamine (50 mg/kg) and xylazine (5 mg/kg) mixture [intraperitoneal (i.p.) injection)] and placed in a stereotaxic apparatus, and then a site in the striatum (coordinate: AP, −0.4 mm; ML, +2.0 mm; DV, −5.0 mm) was selected to inject PBS, NSCs, and iNSCs respectively, according to the grouping. The transplantation of the same amount of cortical NSCs (cNSCs) (*n* = 20) and the injection of the same volume of PBS (vehicle control) (*n* = 20) were used as the control. Cyclosporin A (Sigma-Aldrich) was administered at 10 mg/kg/day subcutaneously beginning at two days before transplantation and continuing daily for 12 weeks after transplantation to all experimental and control groups until the animals were sacrificed for the analysis.

### 4.11. Histology

Mice (*n* = 6/group) were deeply anesthetized via a ketamine (50 mg/kg) and xylazine (5 mg/kg) mixture (i.p.) and transcardially perfused with saline containing 0.5% sodium nitrite and 10 U/mL heparin sulfate. Following perfusion with saline and 4% paraformaldehyde (PFA) in PBS, brains were removed, and forebrain and midbrain blocks were immersion fixed in 4% PFA and cryoprotected in sucrose. Serial coronal sections (30 μm) were cut from bregma −5.2 to +2.2 mm on a cryostat, collected in cryopreservative solution, and stored at −20 °C. Sections were incubated in blocking serum, 10% normal donkey serum in 0.3% tritonX-100 with PBS. Subsequently, sections were incubated in primary antibody solution overnight at 4 °C. Primary antibodies used included TH (Millipore, 1:1000), Tuj-1 (Millipore, 1:1000), NeuN (Millipore 1:2000), GFAP (Millipore, 1:2000), NG2 (Milipore, 1:500), O4 (R&D systems, Minneapolis, MN, USA, 1:1000). Sections were washed in PBS with 0.15% Triton X-100 and incubated in a secondary antibody solution (Alexa^®^ 546 conjugated donkey anti-rabbit antibody, Alexa^®^ 647 conjugated donkey anti-mouse antibody, 1:200) for 1 h at room temperature. Stained sections were mounted on resin-coated slides and dried for 30 min. Slides were then cover-slipped with ProLong^®^ Gold antifade reagent (Invitrogen). Mounted slices were evaluated for fluorescence under settings for 546 and 647 emissions on a confocal microscope (Olympus, Center Valley, PA, USA).

### 4.12. Apomorphine-Induced Rotation

One week after right MFB injection of 6-OHDA, mice were subjected to rotational behavior testing. The animals were injected subcutaneously with 0.5 mg/kg apomorphine hydrochloride (APO, Sigma-Aldrich). The effect of APO on motor asymmetry and the rotation to uninjured side (to the left in our study) in a 60 min period were recorded by two examiners that were blinded to animal states. The mice with contralateral rotations of more than seven cycles per min or total numbers of circling over 420 were considered to validate the pathology model for transplantation at eight weeks. All mice were measured one time every four weeks for 12 weeks after stem cell injection. Total apomorphine-induced rotation numbers were counted two, four, six, and eight weeks after cell injection for transplantation at two weeks.

### 4.13. Statistics

Data were expressed as mean ± standard error of mean (SEM) and analyzed for statistical significance using one-way analysis of variance (ANOVA) followed by Newman-Keuls test as a post-hoc test. Rotation tests were analyzed by two-way repeated measures ANOVA followed by a post hoc least significant difference’s multiple Comparison Test. Null hypotheses of no difference were rejected if *p*-values were less than 0.05. Data analyses were performed with the SPSS software version 20.0 (Armonk, NY, USA).

## 5. Conclusions

In conclusion, our findings that transplanted induced NSCs (iNSCs) into the striatum of 6-hydroxydopamine-injected mice was able to migrate and integrate into the striatum, medial forebrain bundle, and substantia nigra pars compacta, and differentiate into DA neurons. Furthermore, iNSCs improved on motor performance in 6-hydroxydopamine-injected mice. Overall, directly converted iNSCs appear to hold great therapeutic potential to be used as a cell-replacement therapy in PD.

## Figures and Tables

**Table 1:** Primers for RT-qPCR.

Gene Name	Accession Number	Sequence	Annealing Temperature
*Pax6*	NM_001244198	5′-CAAGTTCCCGGGAGTGAACC-3′	60 °C
		5′-TCCACATAGTCATTGGCAGA-3′	
*Sox2*	NM_011443	5′-ACGGCCATTAACGGCACACT-3′	60 °C
		5′-TTTTGCACCCCTCCCAATTC-3′	
*Nes gene*	NM_016701	5′-TCCTGGTCCTCAGGGGAAGA-3′	60 °C
		5′-TCCACGAGAGATACCACAGG-3′	
*Olig2*	NM_016967	5′-ACCACCACGTGTCGGCTATG-3′	60 °C
		5′-TGGTCCAGCTCCCCTTCTTG-3′	
*Blbp*	NM_021272	5′-GGATGGCAAGATGGTCGTGA-3′	60 °C
		5′-TGGGACTCCAGGAAACCAAG-3′	
*Mash1*	NM_008553	5′-CAGAGGAACAAGAGCTGCTG-3′	60 °C
		5′-GATCTGCTGCCATCCTGCTT-3′	
*Col1a1*	NM_007742	5′-CCCTGCCTGCTTCGTGTAAA-3′	60 °C
		5′-TCGTCTGTTTCCAGGGTTGG-3′	
*Thy1*	NM_009382	5′-TTCCCTCTCCCTCCTCCAAGC-3′	60 °C
		5′-TCGAGGGCTCCTGTTTCTCCTT-3′	
*Pdgfrβ*	NM_001146268	5′-CAGGACCTCTGGCTGAAGCA-3′	60 °C
		5′-TCTGGGAGGCAGAAGGGAGAT-3′	
*Gapdh*	NM_008084	5′-CCAATGTGTCCGTCGTGGAT-3′	60 °C
		5′-TGCCTGCTTCACCACCTTCT-3′	

## Abbreviations

PD	parkinson’s disease
DA	dopaminergic
SN	substantia nigra
iNSCs	induced neural stem cells
6-OHDA	6-hydroxydopamine
NSCs	neural stem cells
iPSCs	induced pluripotent stem cells
iDAs	induced DA neurons
mRNA	messenger RNA
MEFs	mouse embryonic fibroblasts
Tuj-1	neuron-specific Class III β-tubulin
GFAP	glial fibrillary acidic protein
MBP	myelin basic protein
MFB	Medial forebrain buddle
SNpC	SN pars compacta
RT-PCR	reverse transcription polymerase chain reaction
TH	tyrosine hydroxylase
DOPAC	3,4-dihydroxyphenylacetic acid
HVA	homovanillic acid
PBS	phosphate-buffered saline
GFP	green fluorescent protein
NeuN	neuronal specific nuclear protein
NG2	neural/glial antigen 2
O4	oligodendrocyte marker O4
